# Cardiorespiratory fitness and muscle strength in offspring conceived through assisted reproductive technologies: results from the Munich heARTerY-study

**DOI:** 10.1007/s00431-025-06261-y

**Published:** 2025-06-21

**Authors:** Marie Kramer, Pengzhu Li, Magdalena Langer, Theresa Vilsmaier, Franziska Sciuk, Brenda Kolbinger, André Jakob, Nina Rogenhofer, Robert Dalla-Pozza, Christian Thaler, Nikolaus Alexander Haas, Felix Sebastian Oberhoffer

**Affiliations:** 1 Division of Pediatric Cardiology and Intensive Care, University Hospital, LMU Munich, 81377 Munich, Germany; 2 Division of Gynecological Endocrinology and Reproductive Medicine, Department of Gynecology and Obstetrics, University Hospital, LMU Munich, 81377 Munich, Germany

**Keywords:** Assisted reproductive technologies (ART), Offspring, Cardiorespiratory fitness, Muscle strength

## Abstract

Children conceived through assisted reproductive technologies (ART) potentially display an increased cardiovascular morbidity. Despite cardiorespiratory fitness (CRF) and muscle strength being key indicators of cardiovascular outcomes, they have not been investigated in ART offspring yet. This observational pilot cohort study aimed to evaluate CRF and muscle strength in ART participants and spontaneously conceived controls. Anthropometric variables, diet quality, level of physical activity, and sedentary behavior were evaluated. Participants performed a 6-min walking test (6MWT) and a 20-m shuttle run test (20mSRT). 6MWT distance and the number of archived laps were assessed, the maximal oxygen uptake (V̇O2_max_) was estimated, and pulse rate recovery was calculated. Maximal hand grip strength (HGS) was determined as a marker of muscle strength. Generalized linear models were used to adjust data for age, birthweight, and gestational age. Sixty-seven ART participants and 86 spontaneously conceived peers were included. Both groups did not differ significantly in age (11.3 (IQR 8.1–18.2) vs. 11.9 (IQR 8.7–18.3) years), gender ratio, anthropometric variables, diet quality, level of physical activity and sedentary behavior. The amount of 20mSRT laps (*P*_adj_=0.02), estimated VO2_max_ (45.0 (IQR 37.9–47.1) vs. 45.8 (IQR 43.1–48.0) ml·kg⁻^1^·min⁻^1^, *P*_adj_=0.04), and pulse rate recovery (*P*_adj_=0.03) were significantly lower in ART participants after adjustment. HGS did not differ between groups.

*Conclusion:* This study indicates a significantly lower CRF in ART participants. Significant differences in muscle strength were not demonstrated between groups. Future studies should validate these results by using cardiopulmonary exercise testing for VO2_max_ assessment.

**Supplementary Information:**

The online version contains supplementary material available at 10.1007/s00431-025-06261-y.

**Table:** 

**What is Known:** • *Children conceived through assisted reproductive technologies (ART) potentially display an increased cardiovascular morbidity. Cardiorespiratory fitness (CRF) and muscle strength, despite being major cardiovascular determinants, have not been assessed within the ART population yet*.
**What is New:** • *This observational pilot cohort study describes and compares CRF and muscle strength in ART participants and spontaneously conceived controls*.

## Introduction

Assisted reproductive technologies (ART) refer to fertility treatments, such as in vitro fertilization (IVF), intracytoplasmic sperm injection (ICSI), or gamete intrafallopian transfer (GIFT), involving manipulation of eggs and sperm [1]. In 2019, 3.4 million ART cycles resulted in approximately 660,000 ART deliveries and 750,000 ART newborns [2].

The application of ART may alter the cardiovascular phenotype of offspring and lead to an increased vascular stiffness, arterial hypertension, and lower left ventricular function [3–5]. However, data is ambiguous as recent studies indicated an unaltered cardiovascular function in ART individuals [6, 7]. These discrepancies may reflect differences in study design, age, or evolving ART protocols [8].

Cardiorespiratory fitness (CRF) is a key indicator of cardiovascular and overall health outcomes [9, 10]. In adolescents, CRF can predict future health outcomes, including the early onset of cardiovascular disease [11]. The gold standard for measuring CRF is maximal oxygen uptake (V̇O2_max_, ml·kg⁻^1^·min⁻^1^) [12]. Field-based tests, such as the 20-m shuttle run test (20mSRT), display high criterion-related validity for estimating V̇O2_max_ (rp=0.78–0.95) and are thus commonly used within the pediatric population as they are easily applicable, time-, and cost-effective [13].

Low muscle strength, often measured by grip strength, is another indicator of cardiovascular disease and -death [14]. For the general population, greater muscle strength in youth is linked with lower cardiovascular risk during adulthood [15].

CRF and muscle strength, despite being major cardiovascular determinants, have not been assessed within the ART population yet. This observational pilot cohort study aimed to compare CRF and muscle strength between ART participants and spontaneously conceived controls.

## Methods

### Ethical statement

The Munich heARTerY-study (Assisted Reproductive Technologies and their effects on heart and arterial function in Youth) was approved on the 27th of December 2020 by the local Ethics Committee (protocol number: 20–0844) and adhered to the ethical guidelines of the Declaration of Helsinki. Written informed consent was obtained from all participants. For underage participants, additional consent was provided by parents or legal guardians.

### Study design and study population

This single-center observational cohort study was conducted at the University Hospital, LMU Munich (Munich, Germany). Data was collected prospectively between May 2021 and March 2022. Healthy individuals without known cardiovascular conditions or other factors affecting CRF or muscle strength were included. Participants aged 4 to 26 years, representing key developmental stages, were examined during a single visit by five trained and non-blinded investigators. From 650 invited ART individuals, 70 participated; after exclusion (e.g., due to T-cell lymphoma, history of heart surgery, incomplete data assessment), 67 ART participants (39 female, 28 male) were analyzed (Figure S1). Eighty-six age- and sex-matched spontaneously conceived controls (44 females, 42 males) were recruited via public calls.

### Assessment of anthropometric variables

Bodyweight (kg), height (cm), and body mass index (BMI, kg/m^2^) were calculated. Standard weight classification was used for adult participants based on absolute BMI values [16]. For minor participants (<18 years of age), BMI percentiles provided by Kromeyer-Hauschild et al. were used for weight classification [17]. Waist circumference (cm) and hip circumference (cm) were measured using a non-elastic, soft plastic measuring tape with participants standing in a relaxed, upright position. The waist-hip ratio was calculated.

### Medical history, course of pregnancy and birth, and maternal level of education

Medical history, smoking status, and regular medication use were recorded. Data on the course of pregnancy and birth was obtained from clinical records and standardized parent interviews, including birth weight (g), gestational age (weeks), maternal age at birth (years), case of multiple pregnancy, maternal BMI at conception (kg/m^2^), the presence of gestational diabetes, and maternal blood pressure during pregnancy ≥140/90 mmHg. Maternal level of education was defined based on the German education system: no school leaving qualification [0], lower secondary school leaving certificate [1], intermediate secondary school leaving certificate [2], general qualification for university entrance [3], completed apprenticeship [4], and completed university degree [5].

### Adherence to the Mediterranean diet, physical activity and sedentary behavior

Adherence to the Mediterranean diet, physical activity, and sedentary behavior were assessed via self-report questionnaires. Adults completed the validated German version of the 14-item Mediterranean diet assessment tool (MEDAS) to evaluate adherence to the Mediterranean diet [18, 19]. Minors completed the validated Mediterranean diet quality index for children and adolescents (KIDMED) test, which was translated into German [20]. This test has not been validated yet in a German population. A score ≥8 was considered high adherence to the Mediterranean diet for both questionnaires [18, 20].

Adults completed the validated German version of the Global Physical Activity Questionnaire provided by the World Health Organization (WHO) to evaluate the level of physical activity [21]. Total and recreational metabolic-equivalent-(MET)-minutes per week as well as muscle strengthening activities per week were assessed [21]. Following WHO guidelines, minors reported daily time spent in moderate-to-vigorous physical activity and weekly frequency of vigorous, muscle- or bone-strengthening exercises [22]. Minors and adults reported daily sedentary time (min) [21]. In all study participants, picture cards were presented for each activity type [21, 22].

### Assessment of cardiorespiratory fitness

The 6-min walking test (6MWT, m) was conducted in accordance with the American Thoracic Society guidelines [23].

For the evaluation of aerobic capacity, participants underwent a 20mSRT (Progressive Aerobic Cardiovascular Endurance Run (PACER) Test, FitnessGram, USA). The 20mSRT was executed according to the manufacturer’s instructions [24]. Participants ran between 20 m-spaced lines, starting at a speed of 8.5 km/h [25]. Each minute, the speed increased by 0.5 km/h. The test was stopped if participants reached exhaustion or failed to complete two consecutive laps within the required time limit [25]. The number of achieved laps was assessed for each participant. Using formulas established by Matsuzaka et al. [25], V̇O2_max_ was estimated for adults as:

$$\dot VO2_{max}\;(ml\cdot kg^{-1}\cdot min^{-1})\;=\;42.4\;-\;(2.85\;\times\;gender)\;-\;(0.488\;\times\;BMI)\;+\;(0.247\;\times\;laps)$$

and for minors as:

$$\dot VO2_{max}\;(ml\cdot kg^{-1}\cdot min^{-1})\;=\;61.1\;-\;(2.20\;\times\;gender)\;-\;(0.462\;\times\;age)\;-\;(0.862\;\times\;BMI)\;+\;(0.192\;\times\;laps)$$

Gender (male=0, female=1) and age (years), BMI (kg/m^2^) were included as input variables.

Before completing the 20mSRT, participants were seated for ≥5 min. Pulse rate (PR, bpm) was measured using a conventional pulse oximeter (Connex Spot Monitor, Welch Allyn, USA) placed on the right index finger only after a stable and valid waveform had been maintained for ≥15 s. Systolic (SBP, mmHg) and diastolic blood pressure (DBP, mmHg) were measured once at the right upper arm using a conventional blood pressure device (Connex Spot Monitor, Welch Allyn, USA). Cuff sizes were chosen based on the circumference of the right upper arm. Following completion of the 20mSRT, participants were seated again, and PR was recorded 1, 5, and 10 min post-exercise to assess relative PR reduction (%), a marker of heart rate (HR) recovery.

### Assessment of muscle strength

Hand grip strength (HGS, kg) of the self-reported dominant hand was measured as a marker of muscle strength [26]. The hand dynamometer (JAMAR Hydraulic Hand Dynamometer, Lafayette Instrument Company, USA) was calibrated according to the manufacturer’s instructions. The instrument was manually adjusted to comfortably match the participant’s grip span. Participants were instructed to sit in a comfortable position, with the shoulder adducted and neutrally rotated. The elbow was flexed to 90°, and the forearm as well as the wrist were maintained in a neutral position. In this study, maximal HGS was measured three times, with a 1-min rest interval between each measurement. The highest value was recorded for analysis.

### Statistical analysis

As this was an observational pilot cohort study, prior power analysis was not feasible. Normality of continuous parameters was tested using the Kolmogorov-Smirnov and the Shapiro-Wilk test. Normally distributed data is presented as mean ± SD and non-normally distributed data as median (IQR). In case of normal distribution, the unpaired *t*-test was used. For non-normally distributed continuous variables, the Mann-Whitney *U* test was utilized. The chi-square test was applied to compare nominal data. Generalized linear models were used to adjust data on CRF as well as muscle strength for age, birthweight, and gestational age. Missing data was not imputed. All analyses used available data only. A *P*<0.05 was regarded as statistically significant. SPSS 29 (IBM, USA) was utilized for data analysis. Data analysis was performed by one investigator.

## Results

### Patients’ characteristics

The final analysis included 67 ART participants (50 ICSI, 16 IVF, 1 GIFT) and 86 controls.

Within the ART group, one participant displayed long QT syndrome, another with a bicuspid aortic valve, one with a possible history of myocarditis, one with hypothyroidism, and one displayed a history of hypercholesterolemia. Four ART participants were on oral contraceptives, one was on L-Thyroxine, and one was on methylphenidate. Within the control group, six participants took oral contraceptives, one participant took bisoprolol due to chronic migraines, and one took methylphenidate.

The ART group and the control group did not differ significantly in age, gender ratio, bodyweight, height, BMI, weight classification, waist-hip ratio, smoking status, maternal BMI at conception, prevalence of gestational diabetes, prevalence of maternal blood pressure ≥ 140/90 mmHg, or maternal educational level (Table 1). Birth weight, birth height, and gestational age were significantly lower in ART participants (*P*<0.001) (Table 1). Within the ART group, maternal age at birth as well as the prevalence of multiple pregnancies were significantly higher (*P*<0.001) (Table 1).

**Table 1:** Patients’ characteristics

Variable	ART (*n*=67)	Control (*n*=86)	*p*-value
Age (years)	11.3 (8.1–18.2)	11.9 (8.7–18.3)	0.43
Female (*n* (%))	39 (58.2)	44 (51.1)	0.39
Bodyweight (kg)	38.7 (23.4–58.8)	42.7 (29.1–59.3)	0.25
Height (cm)	145.0 (125.0–167.0)	157.0 (133.4–170.3)	0.11
BMI (kg/m^2^)	16.8 (15.1–20.8)	17.7 (15.4–21.1)	0.49
Weight classification			
Underweight (*n* (%))	4 (6.0)	6 (7.0)	1
Normal weight (*n* (%))	58 (86.6)	74 (86.1)	
Overweight (*n* (%))	5 (7.4)	6 (6.9)	
Waist-hip ratio	0.9 ± 0.1	0.9 ± 0.1	0.22
Smoking (*n* (%))	3 (4.5)	2 (2.3)	0.65
Course of pregnancy and birth			
Birth weight (g)^1^	2990 (2405–3243)	3440 (3210–3670)	<0.001^***^
Birth height (cm)^2^	50.0 (48.0–52.0)	52.0 (50.0–54.0)	<0.001^***^
Gestational age (weeks)^3^	38.0 (36.0–40.0)	39.0 (38.0–40.0)	<0.001^***^
Maternal age at birth (years)^4^	35.4 ± 3.7	33.1 ± 4.1	<0.001^***^
Multiple pregnancy (n (%))	21 (31.3)	2 (2.3)	<0.001^***^
Maternal BMI at conception (kg/m^2^)^5^	22.5 (20.6–24.7)	21.4 (20.3–22.7)	0.09
Gestational diabetes (*n* (%))^6^	3 (5.3)	3 (4.2)	1
Maternal blood pressure during pregnancy ≥ 140/90 mmHg (*n* (%))^7^	0 (0)	3 (6.4)	0.29
Maternal educational level^8^	4.0 (3.0–5.0)	5.0 (4.0–5.0)	0.22

*ART* assisted reproductive technologies, *BMI* body mass index. ^1^65 ART participants and 79 control participants were included in the analysis. ^2^63 ART participants and 79 control participants were included in the analysis. ^3^62 ART participants and 77 control participants were included in the analysis. ^4^66 ART participants were included in the analysis. ^5^47 ART participants and 61 control participants were included in the analysis. ^6^57 ART participants and 71 control participants were included in the analysis. ^7^28 ART participants and 47 control participants were included in the analysis. ^8^45 ART participants and 52 control participants were included in the analysis. Maternal educational level was assessed according to the German educational system: no school leaving qualification [0], lower secondary school leaving certificate [1], intermediate secondary school leaving certificate [2], general qualification for university entrance [3], completed apprenticeship [4], completed university degree [5]. Data is presented as mean ± SD for normally distributed parameters and as median (IQR) for non-normally distributed parameters. Nominal data is presented as *n* (%). ***P<0.001

### Adherence to the Mediterranean diet, level of physical activity, and sedentary behavior

The groups showed no significant differences in adherence to the Mediterranean diet, level of physical activity, or sedentary behavior (Table 2).

**Table 2:** Adherence to the Mediterranean diet, level of physical activity, and sedentary behavior

Variable	ART (*n*=67)	Control (*n*=86)	*p*-value
Adult study participants	*n*=17	n=22	
MEDAS	6.0 ± 2.4	7.3 ± 1.7	0.06
Total METs (min/week)	5004 ± 4293	4440 ± 2411	0.63
Recreational METs (min/week)	840 (510–4440)	1600 (720–2790)	0.87
Muscle strengthening activities (times/week)	1.0 (0–2.0)	2.0 (0–3.0)	0.37
Sedentary behaviour (min/day)	450 ± 159	409 ± 139	0.40
Minor study participants	*n*=50	*n*=64	
KIDMED	6.2 ± 2.3	6.9 ± 2.1	0.11
Moderate and/or vigorous physical activities (min/day)^1^	90 (60–128)	90 (60–120)	0.26
Vigorous, muscle strengthening and/or bone strengthening activities (times/week)	3.0 (2.0–5.0)	3.0 (2.0–4.0)	0.99
Sedentary behavior (min/day)^2^	360 (270–420)	420 (300–480)	0.13

*ART* assisted reproductive technologies, *MEDAS* Mediterranean diet adherence score, *MET* metabolic-equivalent, *KIDMED* Mediterranean diet quality index for children and adolescents. ^1^49 ART participants and 63 control participants were included in the analysis. ^2^49 ART participants were included in the analysis. Data is presented as mean ± SD for normally distributed parameters and as median (IQR) for non-normally distributed parameters

### Cardiorespiratory fitness and muscle strength

Sixty-six ART participants and 86 controls were included for the analysis of 6MWT distance and maximal hand grip strength. 20mSRT analysis included 63 ART participants and 81 spontaneously conceived controls. Four ART participants and 5 control participants were excluded from 20mSRT analysis due to various reasons (e.g., musculoskeletal injury, the lack of motivation, previous health conditions).

ART participants achieved a significantly lower 6MWT distance (*P*=0.02), a significantly lower amount of 20mSRT laps (*P*<0.001), and a significantly lower estimated VO2_max_ (*P*=0.02) (Table 3). While absolute PR did not differ between groups, ART study participants displayed a significantly lower PR recovery 5 min (*P*=0.01) and 10 min post-testing (*P*=0.02) (Table 3). Maximal HGS did not show significant difference between groups (Table 3). After adjustment for age, birthweight, and gestational age, 20mSRT laps (*P*=0.02), estimated VO2_max_ (*P*=0.04) as well as PR recovery 5 min post-testing (*P*=0.03) remained significantly lower in the ART group. However, 6MWT distance and PR recovery 10 minutes post-testing did not differ significantly between both groups anymore. SBP was significantly lower in ART participants after adjustment (*P*=0.03).

**Table 3:** Cardiorespiratory fitness and muscle strength

Variable	ART (*n*=66)	Control (*n*=86)	*p*-value	Adjusted *p*-value
6MWT distance (m)	643.6 ± 99.7	683.6 ± 107.0	0.02*	0.08
20mSRT^1^				
Laps	22.0 (10.0–31.0)	32.0 (15.0–50.0)	<0.001***	0.02*
Estimated V̇O2_max_ (ml·kg⁻^1^·min⁻^1^)	45.0 (37.9–47.1)	45.8 (43.1–48.0)	0.02*	0.04*
SBP pre-testing (mmHg)	112.1 ± 13.2	115.5 ± 11.2	0.09	0.03*
DBP pre-testing (mmHg)	69.7 ± 8.6	69.8 ± 8.8	0.94	0.99
Pulse rate pre-testing (bpm)	83.1 ± 11.6	81.4 ± 16.2	0.47	0.59
Pulse rate 1 min post-testing (bpm)	137.7 ± 23.3	140.9 ± 21.9	0.39	0.45
Pulse rate 5 min post-testing (bpm)	111.5 ± 11.4	108.9 ± 12.8	0.21	0.08
Pulse rate 10 min post-testing (bpm)^2^	109.0 ± 12.7	106.2 ± 10.0	0.14	0.07
Pulse rate reduction 5 min post-testing (%)	18.1 (11.5–24.4)	24.3 (15.1–28.5)	0.01*	0.03*
Pulse rate reduction 10 min post-testing (%)^2^	21.1 (12.8–28.1)	25.6 (15.4–32.2)	0.02*	0.19
Maximal HGS (kg)	18.0 (11.8–32.0)	20.0 (14.0–32.5)	0.19	0.95
Female (kg)	*n*=38	*n*=44		
	19.0 (11.3–30.0)	21.5 (14.8–31.5)	0.36	0.92
Male (kg)	*n*=28	*n*=42		
	18.0 (13.5–32.5)	20.0 (14.5–38.8)	0.39	0.77

*ART* assisted reproductive technologies, *6MWT* 6-min walking test, *20mSRT* 20-m shuttle run test, *SBP* systolic blood pressure, *DBP* diastolic blood pressure, *VO2*_*max*_, maximal oxygen uptake, *HGS* hand grip strength. ^1^63 ART participants and 81 control participants were included in the analysis. ^2^63 ART participants and 80 control participants were included in the analysis. Data is presented as mean ± SD for normally distributed parameters and as median (IQR) for non-normally distributed parameters. Generalized linear models were used to adjust for age, birthweight, and gestational age. *P<0.05, ***P<0.001

## Discussion

This observational cohort study compared CRF and muscle strength between 67 ART individuals and 86 age- and sex-matched spontaneously conceived peers. No significant differences were observed in anthropometric variables, adherence to the Mediterranean diet, physical activity, or sedentary behavior. ART individuals displayed a significantly lower CRF, visualized by a significantly lower 6MWT distance, amount of achieved 20mSRT laps, estimated VO2_max_, and PR recovery. However, the clinical relevance is uncertain, as differences in estimated VO2 _max_ were marginal, and both groups exhibited median VO2_max_ values within the normal age-related range [27].

Following adjustment for age, birthweight, and gestational age, differences in 6MWT distance were no longer significant. HGS, a marker of overall muscle strength, did not differ significantly between groups.

### Previous findings and underlying mechanisms

#### Cardiovascular morbidity

Data on the long-term cardiovascular morbidity in ART offspring is sparse and inconsistent. A general vascular dysfunction in young ART participants is presumed as some studies have demonstrated a lower endothelial function, higher blood pressure levels, and an increased arterial stiffness and carotid intima-media thickness [3–5]. Additionally, the literature indicates a significantly decreased left ventricular function within the ART cohort [4, 28–30].

In contrast, recent studies do not indicate an altered vascular as well as metabolic function in young ART participants (age range: 4–35 years) [6, 7, 31, 32]. In line with this, the current study demonstrated a significantly lower SBP in ART participants after the adjustment for age, birth weight, and gestational age. Further, large ART cohort studies could not demonstrate an elevated risk for the development of cardiovascular diseases and hospitalization due to cardiovascular diseases [33, 34].

Differences in cardiovascular morbidity among ART individuals may be attributed to ethnic and methodological variations, differences in sample sizes and age ranges, as well as evolving ART protocols over time [4, 5, 8]. The potentially increased cardiovascular morbidity, as postulated by some authors, may explain the significantly lower CRF of ART participants demonstrated in this study [35, 36].

#### Lifestyle factors and training status

Although based on recall data, no significant differences were observed between groups in adherence to the Mediterranean diet, level of physical activity, or sedentary behavior. Recently published data of our working group demonstrated unaltered HbA1c and blood lipid levels in ART youth [7]. While both groups did not differ significantly in BMI, weight classification, or waist-hip ratio, a significantly higher body fat percentage was demonstrated in an earlier publication of our working group for the same ART cohort [7]. Several studies have demonstrated a negative correlation between body fat percentage and VO2_max_ in young adults and athletes [37, 38]. Relative PR rate reduction was significantly lower in ART participants compared to controls suggesting a lower PR recovery. In literature, PR recovery reflects cardiorespiratory training status and is considered a major predictor for mortality [39]. As suggested by Mizrak et al., ART individuals may display sympathetic predominance visualized by a significantly reduced HR variability and vagal modulation [40]. As HR reduction post-exercise is primarily driven by parasympathetic reactivation, the postulated sympathetic predominance in ART individuals might negatively influence PR recovery [41].

#### Pregnancy complications and perinatal risk factors

ART is associated with pregnancy complications and adverse perinatal outcomes [42]. In this study, no significant differences were observed between ART participants and controls in maternal BMI at conception, the prevalence of gestational diabetes, or maternal blood pressure ≥140/90 mmHg during pregnancy. However, ART participants presented with a significantly lower birth weight, birth height, and gestational age. Prematurity, a known risk factor for elevated cardiovascular and pulmonary morbidity, is linked with a reduced VO2_max_, possibly explaining the significantly lower CRF in ART individuals [43]. In this study, 6MWT distance displayed no significant differences between ART individuals and controls when adjusted for age, birth weight, and gestational age. This underlines the potential impairment of CRF due to perinatal risk factors in ART offspring.

##### Infertility, oxidative stress, and epigenetic changes

Parental infertility is linked with an increased cardiovascular morbidity [44, 45]. Potentially, infertile couples that seek ART treatment pass down specific cardiovascular risk factors to their offspring. As experimental studies indicate, ART is thought to be associated with an increased level of oxidative stress due to cryopreservation, culture media, the absence of natural antioxidant mechanisms, and pH and temperature fluctuations [46]. Moreover, female infertility, advanced maternal age, pregnancy complications, and perinatal risk factors might additionally elevate oxidative stress levels, as demonstrated by studies in mice and in humans [8, 42, 46]. High levels of oxidative stress may lead to epigenetic changes and thus negatively influence cardiovascular development and morbidity [46]. A recent study by Mertens et al. was able to show that ART individuals present with an altered mitochondrial DNA genotype due to de novo mutagenesis linked with maternal aging as well as ovarian stimulation-induced oocyte cohort size [47]. Possible long-term health consequences, including the influence of these mitochondrial genetic changes on CRF, need to be established in the future [36, 47].

### Strengths and limitations

#### Study design

In this single-center observational study, special emphasis was put on matching both groups (e.g., age, gender ratio, anthropometric variables, adherence to the Mediterranean diet, level of physical activity, and sedentary behavior). CRF and muscle strength data was adjusted for age, gestational age, and birthweight. As this was a non-blinded study and participants were examined by multiple investigators, participation bias, assessor bias, and interobserver variability cannot be ruled out. The wide age range and inclusion of individuals with adverse perinatal conditions may have negatively affected the outcomes. The study did not account for confounders such as ART type (e.g., IVF, ICSI), parental health, or socioeconomic status. As ART offspring may present with various comorbidities and risk factors that could influence CRF and muscle strength, larger multi-center studies are required to adjust for these confounders.

#### Methodology

Pregnancy and birth data was collected retrospectively from medical records and parental interviews. Due to missing or insufficiently documented records, data was incomplete in some participants. Diet quality, physical activity, and sedentary behavior were assessed via recall-based questionnaire. The 20mSRT was applied to evaluate CRF, a valid, easily applicable, time-, and cost-effective method to estimate VO2_max_ [13]. VO2_max_ was estimated using formulas by Matsuzaka et al. [25], which have shown low bias, minimal estimate range variation, and strong correlation values [48]. The standard errors of the estimate for VO2_max_ range between 3 and 3.4 ml·kg⁻^1^·min⁻^1^ for formulas established by Matsuzaka et al. [25]. This could affect the findings of the current study as median VO2_max_ differed only by 0.8 ml·kg⁻^1^·min⁻^1^ between both groups. As formulas by Matsuzaka et al. are based on a Japanese cohort aged 8 to 23 years, they might not be fully applicable for the current study cohort [25]. Further, the relatively high starting speed of the 20mSRT might be too high for minors ultimately leading to a lower test validity [13]. Future research should consider CPET for direct VO2_max_ measurement.

## Conclusion

This study indicates a significantly lower CRF in ART participants compared to spontaneously conceived controls. Significant differences in muscle strength were not demonstrated between ART participants and controls. Future research should consider CPET for direct VO2_max_ measurement. Larger, multi-center follow-up studies are required for precise cardiorespiratory risk stratification of the ART cohort.

## Supplementary Information

Below is the link to the electronic supplementary material.

Supplementary file1 (DOCX 30 KB)

## Data Availability

Not applicable.

## Abbreviations

ART: Assisted reproductive technologies
BMI: Body mass index
CPET: Cardiopulmonary exercise testing
CRF: Cardiorespiratory fitness
DBP: Diastolic blood pressure
GIFT: Gamete intrafallopian transfer
HGS: Hand grip strength
HR: Heart rate
ICSI: Intracytoplasmic sperm injection
IVF: In vitro fertilization
KIDMED: Mediterranean diet quality index for children and adolescents
MEDAS: Mediterranean diet assessment tool
MET: Metabolic equivalent
PACER: Progressive Aerobic Cardiovascular Endurance Run
PR: Pulse rate
SBP: Systolic blood pressure
V̇O_2max_: Maximal oxygen uptake
WHO: World Health Organization
6MWT: 6-min walking test
20mSRT: 20-m shuttle run test
