# Supplementary material for: Cardiorespiratory fitness and muscle strength in offspring conceived through assisted reproductive technologies: results from the Munich heARTerY-study
Source: Eur J Pediatr. 2025 Jun 21;184(7):431. doi: 10.1007/s00431-025-06261-y (PMC12182500; doi:10.1007/s00431-025-06261-y)
Supplement: Supplementary file 1 — Supplementary file1 (DOCX 30 KB) [file 431_2025_6261_MOESM1_ESM.docx]

Figure S1 Flow Chart visualizing Enrollment of Individuals Conceived Through Assisted Reproductive Technologies (ART)

Excluded (n = 3)

- History of T-cell lymphoma = 1
- History heart surgery = 1
- Incomplete data

Final amount of ART individuals included
(n = 67)

No response / decline in participation (n = 580)

ART individuals contacted

(n = 650)

**Screening**

**Included**

**Identification**

ART individuals examined

(n = 70)
